# Chest physiotherapy during immediate postoperative period among patients undergoing upper abdominal surgery: randomized clinical trial

**DOI:** 10.1590/S1516-31802008000500005

**Published:** 2008-09-04

**Authors:** Roberta Munhoz Manzano, Celso Ricardo Fernandes de Carvalho, Beatriz Mangueira Saraiva-Romanholo, Joaquim Edson Vieira

**Keywords:** Physical therapy (specialty), Postoperative period, Surgery, Anesthesia recovery period, Spirometry, Fisioterapia, Período pós-operatório, Cirurgia, Período de recuperação da anestesia, Espirometria

## Abstract

**CONTEXT AND OBJECTIVE:**

Abdominal surgical procedures increase pulmonary complication risks. The aim of this study was to evaluate the effectiveness of chest physiotherapy during the immediate postoperative period among patients undergoing elective upper abdominal surgery.

**DESIGN AND SETTING:**

This randomized clinical trial was performed in the post-anesthesia care unit of a public university hospital.

**METHODS:**

Thirty-one adults were randomly assigned to control (n = 16) and chest physiotherapy (n = 15) groups. Spirometry, pulse oximetry and anamneses were performed preoperatively and on the second postoperative day. A visual pain scale was applied on the second postoperative day, before and after chest physiotherapy. The chest physiotherapy group received treatment at the post-anesthesia care unit, while the controls did not. Surgery duration, length of hospital stay and postoperative pulmonary complications were gathered from patients' medical records.

**RESULTS:**

The control and chest physiotherapy groups presented decreased spirometry values after surgery but without any difference between them (forced vital capacity from 83.5 ± 17.1% to 62.7 ± 16.9% and from 95.7 ± 18.9% to 79.0 ± 26.9%, respectively). In contrast, the chest physiotherapy group presented improved oxygen-hemoglobin saturation after chest physiotherapy during the immediate postoperative period (p < 0.03) that did not last until the second postoperative day. The medical record data were similar between groups.

**CONCLUSIONS:**

Chest physiotherapy during the immediate postoperative period following upper abdominal surgery was effective for improving oxygen-hemoglobin saturation without increased abdominal pain. Breathing exercises could be adopted at post-anesthesia care units with benefits for patients.

**CLINICAL TRIAL REGISTRATION NUMBER:**

NCT00596778

## RESUMO

**CONTEXTO E OBJETIVO:**

Cirurgias abdominais podem aumentar o risco de complicações pulmonares. O objetivo foi avaliar a eficácia da fisioterapia respiratória precoce no pós-operatório imediato em pacientes submetidos a cirurgia abdominal alta.

**TIPO DE ESTUDO E LOCAL:**

O estudo foi do tipo ensaio clínico aleatório, realizado na sala de recuperação pós-anestésica de um hospital público universitário.

**MÉTODO:**

31 adultos foram selecionados aleatoriamente em dois grupos, controle (n = 16) e grupo de fisioterapia respiratória (n = 15). Espirometria, oximetria de pulso e anamnese foram realizadas no pré-operatório e no segundo dia pós-operatório. A escala visual analógica foi aplicada no segundo dia pós-operatório e antes e depois da fisioterapia respiratória. O grupo da fisioterapia respiratória recebeu fisioterapia na sala de recuperação pós-anestésica e o grupo controle, não. Tempo cirúrgico, tempo de internação e as complicações pulmonares pós-operatórias foram analisados nos prontuários dos pacientes.

**RESULTADOS:**

Os grupos apresentaram uma diminuição dos valores espirométricos após a cirurgia, porém, não houve diferença entre eles (capacidade vital forçada, CVF 83.5 ± 17.1% *versus* 62.7 ± 16.9% grupo controle pré *versus* pós-operatório e 95.7 ± 18.9% *versus* 79.0 ± 26.9% grupo fisioterapia). Em contraste, o grupo da fisioterapia respiratória apresentou uma melhora na saturação de oxi-hemoglobina após a fisioterapia no pós-operatório imediato (p < 0,03), mas esta não se manteve até o segundo dia pós-operatório.

**CONCLUSÕES:**

A fisioterapia respiratória é efetiva na melhora da saturação de oxi-hemoglobina em pacientes observados na sala de recuperação pós-anestésica em cirurgia abdominal alta, e não provoca dor durante os exercícios.

## INTRODUCTION

Surgery and general anesthesia directly affect the respiratory system.^1-3^ Upper abdominal surgery alters postoperative pulmonary function, as observed by impairment of lung volumes such as total lung capacity,^1^ vital capacity and tidal volume. It also reduces the efficiency of efforts to cough for as long as one week. There are also falls in oxygen arterial pressure and in oxygen-hemoglobin saturation.^1,4^ Postoperative pulmonary complications occur after upper abdominal surgery at a rate ranging from 6 to 70% of patients, depending on the criteria for defining them.^1,5-7^ They may include atelectasis, pneumonia or hypoxemia, among others.^3,7,8^

Breathing and chest wall physiotherapy have been used to prevent atelectasis.^9^ Respiratory exercises during hospitalization has been shown to improve respiratory muscle strength, oxygenation, coughing mechanism, chest wall mobility and lung ventilation, as well as decreasing respiratory work and preventing postoperative pulmonary complications.^10-12^ The effects of different chest physiotherapy regimens have been evaluated among high-risk postoperative patients and none of them could be considered highly satisfactory with regard to preventing such complications.^13^ On the other hand, preoperative chest physiotherapy reduced the incidence of postoperative pulmonary complications and improved mobilization and oxygen-hemoglobin saturation after major abdominal surgery.^14^

The hypothesis of this study was that chest physiotherapy during the immediate postoperative period among patients who had undergone upper abdominal surgery would improve the postoperative results or prevent postoperative pulmonary complications.

## OBJECTIVE

The aim of the present study was to evaluate the effectiveness of chest physiotherapy during the immediate postoperative period among patients undergoing elective upper abdominal surgery, administered during anesthesia recovery at the post-anesthesia care unit (PACU).

## METHODS

This randomized clinical trial evaluated patients who had been scheduled for elective upper abdominal surgery at a university hospital. Thirty-one consecutive patients were selected from the schedule for abdominal surgery, independent of gender and age. All of them were at the preoperative stage preceding elective upper abdominal surgery.

The procedures that they were about to undergo included hernia repair, gall bladder removal, large bowel removal, exploratory laparotomy or other interventions in the abdominal cavity performed by conventional laparotomy. All of the patients underwent general anesthesia. Patients with an indication for liver transplantation, or who presented aneurysm of any arterial segment, were excluded since these patients stay in the intensive care unit for a longer time during the postoperative period. Patients undergoing videolaparoscopy surgery were also excluded, since this induces smaller changes in the postoperative breathing mechanics than laparotomy does.

Information concerning preoperative and intraoperative procedures and postoperative complications was gathered from the patients' medical records. The participants were allocated into two groups (control and chest physiotherapy) by means of a draw according to a randomization table. Whenever an intensive care unit intervention would be required, the patient would be removed from the protocol. The level of oxygen-hemoglobin saturation measured by noninvasive oximetry was chosen as the main outcome. The secondary outcomes were pain during the chest physiotherapy and spirometry on the second postoperative day.

This study was approved by the hospital's Ethics Committee under protocol number 836/03.

### Statistical methods

A normality test (Kolmogorov-Smirnov) was used to differentiate between parametric and nonparametric data. Differences between and within groups were analyzed by using Student's t-test and one-way repeated measurement analysis of variance (ANOVA). The sample size calculation took into account a minimum mean difference of 2.5% for oximetry, with a standard deviation of 2, test power of 80% and alpha of 0.05, thus resulting in a requirement of 11 patients for each group. Descriptive analysis was performed on all data (means, standard deviations and medians). Student's t-test was also used to compare the variables of length of stay and surgery duration. The pulse oximetry values were compared by means of repeated measurement ANOVA, followed by the Tukey post-hoc test. A statistical package was used for all tests (Sigma Stat for Windows, version 3.11, San José, California, United States).

### Procedures

*Control group:* The patients in the control group were evaluated on the day before surgery and on the second postoperative day. Anamnesis, physical examination, pulse oximetry and spirometric tests were performed before and after surgery (second day). They did not receive any chest physiotherapy. Before and after the spirometry, the patients were asked to subjectively describe their pain using an visual analog pain scale (VAPS), which comprised numbers from 0 (no pain whatsoever) to 10 (worst possible pain).^15,16^ Information regarding the type and duration of surgery and the amounts of painkiller and antibiotic prescriptions was retrieved from the medical records.

*Chest physiotherapy group:* On the day before surgery and on the second postoperative day, the patients in the chest physiotherapy group underwent anamnesis, physical examination, pulse oximetry and spirometric tests. After surgery, while still in the PACU, as soon as the patients achieved a score of 10 on the Aldrete-Kroulik index,^17^ they were evaluated and subjected to one session of chest physiotherapy. The protocol consisted of breathing exercises for 30 minutes and included: passive and localized exercises,^10,12^ deep diaphragmatic breathing^10,12^ and chest wall expansion exercises^12^ (Table 1). The same protocol regarding VAPS and medical records as described above for the control group was applied to the chest physiotherapy group.

Later on (30 days after surgery), all patients in both groups were contacted by telephone and asked about any postoperative pulmonary complications such as coughing, dyspnea, fever, sputum in airways and the need for additional medication.

**Table 1.** Chest physiotherapy exercises that were performed^10,12^

Passive and localized exercises	Localized breathing exercises associated with manual pressure performed by the physiotherapist on patients' chest wall during expiration
Deep diaphragmatic breathing	Slow deep inspiration, asking the patient to expand the diaphragmatic region, followed by slow expiration
Chest expansion exercises	Deep inspiration followed by a three-second pause at maximal inspiratory volume attained, and then slow expiration.

*Spirometry:* The spirometric evaluation, on the second postoperative day, was performed using Koko Spirometer, Pulmonary Data Services, Colorado, United States. The technical procedures and the acceptability and reproducibility criteria were those recommended by the American Thoracic Society.^18^ The following variables were recorded and expressed as body temperature, ambient pressure and water vapor saturation (BTPS) conditions: forced vital capacity (FVC, %) and forced expiratory volume in one second (FEV_1_, %). The predicted normal values were those proposed by Knudson.^19^ The peak expiratory flow rate (PEF, liters/min) was obtained using a peak flow meter.

## RESULTS

All the patients randomly assigned to the treatments were analyzed (intention-to-treat analysis). Before surgery, the patients' anthropometric data were similar between the two groups (control and chest physiotherapy) (Table 2). Surgery duration and incidence of breathing complications did not differ between the groups. One patient in the control group reported an episode of shortness of breath that required medical assistance but presented no further complication. None of the participants received any indication for intensive care unit procedures after discharge from the PACU.

**Table 2.** Patients' preoperative data

	Control group (16)	Chest physiotherapy group (15)
Sex F/M	10 F/6 M	11 F/4 M
Age (years)	50.9 ± 16.6	52.0 ± 11.8
BMI (kg/m^2^)	22.7 ± 4.0	24.5 ± 3.9
Respiratory rate (ipm)	19.8 ± 3.9	20.4 ± 3.4
Heart rate (bpm)	86.8 ± 22.9	75.7 ± 13.8
SpO_2_ (%)	96.6 ± 1.5	96.4 ± 1.9
Surgery duration (in minutes)	229.7 ± 58.9	240.7 ± 50.8

Data are presented as means ± standard deviations. F = female; M = male; BMI = body mass index = weight/height^2^; ipm = inspirations per minute; bpm = beats per minute; SpO_2_(%) = oxygen-hemoglobin saturation (percentage).

The preoperative spirometric values (expressed as a percentage of the predicted values) were similar in the two groups and presented average decreases of 20% in FVC, FEV1 and PEF on the second postoperative day (Table 3).

**Table 3.** Spirometry and peak expiratory flow in the control and chest physiotherapy groups before and after surgery

	Control group			Chest physiotherapy group			Control versus chest physiotherapy	
	pre	post	p	pre	post	p	p - pre	p - post
**FVC **(%)	83.5 ± 17.1	62.7 ± 16.9	**< 0.001**	95.7 ± 18.9	79.0 ± 26.9	**< 0.009**	**0.075**	**0.056**
**FEV**_1_(%)	89.5 ± 18.6	69.6 ± 13.3	**< 0.001**	96.6 ± 18.8	79.9 ± 25.0	**< 0.004**	**0.316**	**0.182**
**FEV**_1_**/FVC**(%)	107.0 ± 13.5	108.9 ± 15.6	**0.460**	101.5 ± 10.6	103.0 ± 11.3	**0.504**	**0.227**	**NA**
**PEF**(liters/min)	363.4 ± 118.9	258.1 ± 85.4	**< 0.001**	388.7 ± 119.6	290.0 ± 99.1	**0.002**	**0.561**	**0.345**

FVC = forced vital capacity; FEV_1_ = forced expiratory volume in one second; PEF = peak expiratory flow; NA = not applicable.

The two groups presented similar levels of pain before surgery and on the second postoperative day Table 4. The group receiving chest physiotherapy presented a lower pain score on the immediate postoperative day. There was no difference considering the use of analgesics for both groups (Table 5).

**Table 4.** Visual analog pain scale

			Chest physiotherapy group		
	Control group	Chest physiotherapy group	Before	After	p
**Preoperative**	3.0 [2.0 - 3.6]	3.0 [1.3 - 4.5]			**0.474**
			2.0 [2.0 - 7.3]	2.0 [2.0 - 6.5]	**0.500**
**Postoperative**	3.0 [2.0 - 3.6]	3.0 [1.3 - 3.0]			**0.550**

Data presented as median and interquartile range [25% – 75%].

**Table 5.** Analgesic use during stay in post-anesthesia care unit for the control and chest physiotherapy groups

	Control group (n = 16)	Chest physiotherapy group (n = 15)
**Tramadol** *	14 (88%)	9 (60%)
**Dipyrone** *	14 (88%)	14 (88%)
**Ketoprofen** *	7 (44%)	5 (33%)
**Morphine** *	1 (6%)	1 (7%)
**Acetaminophen** *	1 (6%)	4 (27%)
		
**Codeine**	none	1 (7%)
**Bupivacaine and Fentanil (PCA)**	1 (6%)	none

* Chi-square = 3,002 (degrees of freedom = 4); p = 0.558. PCA = patient controlled analgesia.

The oxygen-hemoglobin saturations found from preoperative and postoperative measurements were different for the control group (96.6 ± 1.5 versus 95.1 ± 1.9, p = 0.006) and chest physiotherapy group (96.4 ± 1.9 versus 94.7 ± 2.4, p = 0.02) (Table 6). On the other hand, the treatment group showed higher values after physiotherapy while still in the PACU (Table 6). However, this group's oxygen saturation showed lower values on the second postoperative day that were not different from those measured after surgery (93.6 ± 4.3 versus 94.7 ± 2.4, p = 0.70) (Table 7).

**Table 6.** Oxygen-hemoglobin saturation before and after the operation in the control and chest physiotherapy groups

	Control group		p	Chest physiotherapy group		p
	Before surgery	After surgery		Before surgery	After surgery	
**SatO**_2_(%)	96.6 ± 1.5	95.1 ± 1.9	**p = 0.006**	96.4 ± 1.9	94.7 ± 2.4	**p = 0.02**
**mean ± SD**	96.6 ± 1.5			96.4 ± 1.9		**p = 0.71**
		95.1 ± 1.9			94.7 ± 2.4	**p = 0.61**

SD = standard deviation.

**Table 7.** Oxygen-hemoglobin saturation before and after the operation in the chest physiotherapy group

Chest physiotherapy group						
	Before surgery	Before physiotherapy	p	After physiotherapy	After surgery	p
	96.4 ± 1.9	93.6 ± 4.3	**p = 0.033**	96.0 ± 2.6	94.7 ± 2.4	**p = 0.53**
**SatO**_2_(%)	96.4 ± 1.9			96.0 ± 2.6		**p = 0.97**
**mean ± SD**		93.6 ± 4.3		96.0 ± 2.6		**p = 0.02**
		93.6 ± 4.3			94.7 ± 2.4	**p = 0.70**

SD = standard deviation.

## DISCUSSION

The present study shows that chest physiotherapy performed immediately after upper abdominal surgery improves oxygen-hemoglobin saturation without increased pain. Chest physiotherapy has been shown to prevent or even to improve breathing complications such as secretions, atelectasis and pneumonia, using a variety of techniques.^20^ Together with postoperative care, respiratory physiotherapy techniques seem to provide some benefit in reducing pulmonary complications.^1,3,7,20-22^

The postoperative spirometry results presented in this study by the two groups did not show any significant differences. Spirometry as a means of quantifying lung function is controversial. Its best results may not be achieved after abdominal surgery, since patients are unable to perform at their best or even to make a moderate effort to reach total pulmonary capacity or produce maximal forced expirations.^23^ The results from this study may add to other authors' investigations, to suggest that there is no evidence that spirometry has any predictive value with regard to postoperative pulmonary complications other than what is supplied by clinical evaluation, considering the short observation period.^4,7,23-25^

There was no difference in measured pain during the preoperative and postoperative periods for either group, or after physiotherapy. Some patients in the chest physiotherapy group even reported some pain reduction after the exercises. These findings are at odds with the reasoning that mobilization may increase pain intensity after abdominal surgery.^26^ Nonetheless, these same findings are in line with the notion that not only analgesic treatment but also physiotherapy for abdominal and thoracic surgery can reduce the hospital stay and improve recovery.^27^

In the present study, the oxygen-hemoglobin saturation increased after physiotherapy.^14^ It is of interest to notice that, comparing the times before and after physiotherapy, the saturation increased even with the decay on the second day after surgery. Since these values did not last two days, it is reasonable to suggest that patients would benefit from additional chest exercises during and after their PACU stay. We believe that with additional exercises, this oxygen-hemoglobin saturation improvement should last longer, although new studies would be necessary. The topic of physiotherapy exercises during the post-anesthesia care period seems to be quite new in the literature, and it could be reasonable to suggest that this study represents a first report showing oximetry improvement after physiotherapy exercises in the PACU, among non-obese patients. A previous report suggested that Trendelenburg lateral decubitus and bed-flat lateral decubitus positions do not induce clinically significant desaturation among obese patients, but those authors did not use physiotherapy exercises.^28^

Some limitations should be borne in mind regarding this study. This protocol did not envisage any visit to the control group during these patients' PACU stay. Although this could have improved the statistical comparison, it reflected the practice promoted nowadays. Attention should be drawn to the fact that the patients who received chest physiotherapy did not report high levels of pain. Because the protocol did not have the aim of investigate whether physiotherapy during post-anesthesia recovery could induce pain, additional studies would be necessary to address this condition. Considering the controversy in the literature regarding physiotherapy techniques and the new approach showed here for early post anesthetic care, it is reasonable to believe that additional research involving physiotherapy in the PACU ought to bring interesting findings. Finally, with regard to the apparently short duration of high levels of oxygen saturation, additional studies could be undertaken to address the numbers, techniques and intervals of physiotherapy procedures that could be applied to benefit these patients.

## CONCLUSIONS

The results from this study showed that chest physiotherapy during the immediate postoperative period following upper abdominal surgery improved the oxygen-hemoglobin saturation.
